# 5-(4-Methyl­phenyl­sulfon­yl)-1,3-dithiolo[4,5-*c*]pyrrole-2-thione

**DOI:** 10.1107/S1600536812009622

**Published:** 2012-03-14

**Authors:** Ning-Juan Zheng, Bing-Zhu Yin

**Affiliations:** a Key Laboratory of Natural Resources of Changbai Mountain & Functional Molecules (Yanbian University), Ministry of Education, Yanji 133002, People’s Republic of China

## Abstract

The asymmetric unit of the title compound, C_12_H_9_NO_2_S_4_, contains one half-mol­ecule with the N, two S amd four C atoms lying on a mirror plane. The mol­ecule exhibits a V-shaped conformation, with a dihedral angle of 87.00 (7)° between the benzene and dithiol­opyrrole rings. The methyl group was treated as rotationally disordered between two orientations in a 1:1 ratio. In the crystal, weak C—H⋯O hydrogen bonds link the mol­ecules into chains in [010].

## Related literature

For background to the applications and synthesis of pyrrolo-annulated tetra­thia­fulvalenes, see: Becher *et al.* (2004 ▶); Hou *et al.* (2010 ▶). For a related structure, see: Hou *et al.* (2009 ▶). For details of the synthesis, see: Jeppesen *et al.*(2000 ▶). 
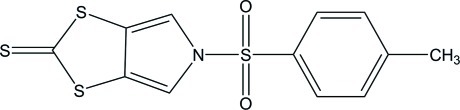


## Experimental

### 

#### Crystal data

C_12_H_9_NO_2_S_4_
*M* *_r_* = 327.44
Monoclinic, 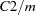

*a* = 15.687 (10) Å
*b* = 10.485 (9) Å
*c* = 8.255 (4) Å
β = 96.19 (3)°
*V* = 1349.9 (16) Å^3^
*Z* = 4
Mo *K*α radiation
μ = 0.70 mm^−1^
*T* = 290 K
0.46 × 0.43 × 0.40 mm

#### Data collection

Rigaku R-AXIS RAPID diffractometer
Absorption correction: multi-scan (*ABSCOR*; Higashi, 1995 ▶) *T*_min_ = 0.740, *T*_max_ = 0.770
6671 measured reflections
1633 independent reflections
1467 reflections with *I* > 2σ(*I*)
*R*_int_ = 0.020

#### Refinement

*R*[*F* ^2^ > 2σ(*F* ^2^)] = 0.029
*wR*(*F* ^2^) = 0.094
*S* = 1.19
1633 reflections
99 parameters
H-atom parameters constrained
Δρ_max_ = 0.31 e Å^−3^
Δρ_min_ = −0.31 e Å^−3^

### 

Data collection: *RAPID-AUTO* (Rigaku, 1998 ▶); cell refinement: *RAPID-AUTO*; data reduction: *CrystalStructure* (Rigaku/MSC and Rigaku, 2002 ▶); program(s) used to solve structure: *SHELXS97* (Sheldrick, 2008 ▶); program(s) used to refine structure: *SHELXL97* (Sheldrick, 2008 ▶); molecular graphics: *SHELXTL* (Sheldrick, 2008 ▶); software used to prepare material for publication: *SHELXL97*.

## Supplementary Material

Crystal structure: contains datablock(s) global, I. DOI: 10.1107/S1600536812009622/cv5254sup1.cif

Structure factors: contains datablock(s) I. DOI: 10.1107/S1600536812009622/cv5254Isup2.hkl

Supplementary material file. DOI: 10.1107/S1600536812009622/cv5254Isup3.cml

Additional supplementary materials:  crystallographic information; 3D view; checkCIF report

## Figures and Tables

**Table 1:** Hydrogen-bond geometry (Å, °)

*D*—H⋯*A*	*D*—H	H⋯*A*	*D*⋯*A*	*D*—H⋯*A*
C3—H3⋯O1^i^	0.93	2.33	3.243 (3)	166

Symmetry code: (i) 
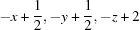
.

**Fig. 1.**
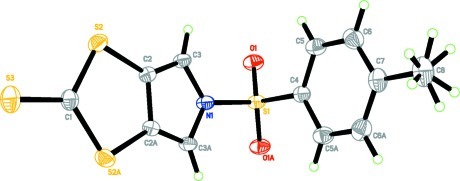
The molecular structure of (I) with the atom numbering. Displacement ellipsoids are drawn at the 30% probalility level [symmetry code (A): x, -y, z].

## supplementary crystallographic information

### Comment

Pyrrolo-annulated tetrathiafulvalenes, an important class of electron-donors, are versatile building blocks in supramolecular and materials chemistry (Becher *et al.*, 2004). As a key precursor to the pyrrolo-annulated tetrathiafulvalenes, 5-Tosyl-5*H*-[1,3]dithiolo[4,5-*c*]pyrrole-2-thione, has attracted great attention (Hou *et al.*, 2010). In this paper, we report the crystal structure of the title compound (I).

The asymmetric unit of (I) contains a half of the molecule situated on a mirror plane (Fig. 1). All bond lengths and angles are in the normal ranges and comparable with the reported ones (Hou *et al.* 2009). Atom N1 has a flattened pyramidal environment with the sum of bond angles of 356.9 (2) °. The benzene ring and dithiolopyrrole ring form a dihedral angle of 87.00 (7) °. In the crystal, the intermolecular C—H···O hydrogen bonds link the molecules into chains along *b* direction.

### Experimental

The title compound was prepared according to the literature (Jeppesen *et al.*, 2000). Single crystals suitable for X-ray diffraction were prepared by slow evaporation a mixture of dichloromethane and petroleum (60–90 °C) at room temperature.

### Refinement

C-bound H-atoms were placed in calculated positions (C—H 0.93 and 0.96 Å) and were included in the refinement in the riding model with *U*_iso_(H) = 1.2 or 1.5 *U*_eq_(C). The methyl group was treated as rotationally disordered between two orientations in a ratio 1:1.

### Crystal data

**Table:**

C_12_H_9_NO_2_S_4_	*F*(000) = 672
*M**_r_* = 327.44	*D*_x_ = 1.611 Mg m^−^^3^
Monoclinic, *C*2/*m*	Mo *K*α radiation, λ = 0.71073 Å
Hall symbol: -C 2y	Cell parameters from 6182 reflections
*a* = 15.687 (10) Å	θ = 3.3–27.5°
*b* = 10.485 (9) Å	µ = 0.70 mm^−^^1^
*c* = 8.255 (4) Å	*T* = 290 K
β = 96.19 (3)°	Block, yellow
*V* = 1349.9 (16) Å^3^	0.46 × 0.43 × 0.40 mm
*Z* = 4	

### Data collection

**Table:**

Rigaku R-AXIS RAPID diffractometer	1633 independent reflections
Radiation source: fine-focus sealed tube	1467 reflections with *I* > 2σ(*I*)
Graphite monochromator	*R*_int_ = 0.020
ω scans	θ_max_ = 27.5°, θ_min_ = 3.3°
Absorption correction: multi-scan (*ABSCOR*; Higashi, 1995)	*h* = −20→16
*T*_min_ = 0.740, *T*_max_ = 0.770	*k* = −13→13
6671 measured reflections	*l* = −10→10

### Refinement

**Table:**

Refinement on *F*^2^	Secondary atom site location: difference Fourier map
Least-squares matrix: full	Hydrogen site location: inferred from neighbouring sites
*R*[*F*^2^ > 2σ(*F*^2^)] = 0.029	H-atom parameters constrained
*wR*(*F*^2^) = 0.094	*w* = 1/[σ^2^(*F*_o_^2^) + (0.053*P*)^2^ + 0.4647*P*] where *P* = (*F*_o_^2^ + 2*F*_c_^2^)/3
*S* = 1.19	(Δ/σ)_max_ = 0.012
1633 reflections	Δρ_max_ = 0.31 e Å^−^^3^
99 parameters	Δρ_min_ = −0.31 e Å^−^^3^
0 restraints	Extinction correction: *SHELXL97* (Sheldrick, 2008), Fc^*^=kFc[1+0.001xFc^2^λ^3^/sin(2θ)]^-1/4^
Primary atom site location: structure-invariant direct methods	Extinction coefficient: 0.0106 (12)

### Special details

**Table:**

Experimental. (See detailed section in the paper)
Geometry. All e.s.d.'s (except the e.s.d. in the dihedral angle between two l.s. planes) are estimated using the full covariance matrix. The cell e.s.d.'s are taken into account individually in the estimation of e.s.d.'s in distances, angles and torsion angles; correlations between e.s.d.'s in cell parameters are only used when they are defined by crystal symmetry. An approximate (isotropic) treatment of cell e.s.d.'s is used for estimating e.s.d.'s involving l.s. planes.
Refinement. Refinement of *F*^2^ against ALL reflections. The weighted *R*-factor *wR* and goodness of fit *S* are based on *F*^2^, conventional *R*-factors *R* are based on *F*, with *F* set to zero for negative *F*^2^. The threshold expression of *F*^2^ > σ(*F*^2^) is used only for calculating *R*-factors(gt) *etc*. and is not relevant to the choice of reflections for refinement. *R*-factors based on *F*^2^ are statistically about twice as large as those based on *F*, and *R*- factors based on ALL data will be even larger.

### Fractional atomic coordinates and isotropic or equivalent isotropic displacement parameters (Å^2^)

**Table:**

	*x*	*y*	*z*	*U*_iso_*/*U*_eq_	Occ. (<1)
C1	0.40151 (14)	0.0000	0.3618 (3)	0.0381 (5)	
C2	0.33159 (9)	0.06779 (14)	0.61983 (17)	0.0328 (3)	
C3	0.29981 (10)	0.10896 (15)	0.75702 (18)	0.0360 (3)	
H3	0.2918	0.1931	0.7875	0.043*	
C4	0.11420 (15)	0.0000	0.8848 (2)	0.0352 (4)	
C5	0.07509 (12)	0.11519 (17)	0.8411 (2)	0.0451 (4)	
H5	0.1013	0.1919	0.8736	0.054*	
C6	−0.00330 (12)	0.1139 (2)	0.7486 (2)	0.0509 (4)	
H6	−0.0298	0.1908	0.7179	0.061*	
C7	−0.04379 (16)	0.0000	0.7000 (3)	0.0472 (6)	
C8	−0.12828 (19)	0.0000	0.5964 (3)	0.0635 (8)	
H8A	−0.1210	−0.0344	0.4910	0.095*	0.50
H8B	−0.1495	0.0858	0.5846	0.095*	0.50
H8C	−0.1685	−0.0514	0.6473	0.095*	0.50
N1	0.28154 (13)	0.0000	0.8431 (2)	0.0356 (4)	
O1	0.23189 (8)	0.11804 (11)	1.07477 (13)	0.0442 (3)	
S1	0.21652 (4)	0.0000	0.99190 (6)	0.03409 (18)	
S2	0.37336 (3)	0.14002 (4)	0.45643 (5)	0.04288 (17)	
S3	0.45063 (5)	0.0000	0.19690 (8)	0.0555 (2)	

### Atomic displacement parameters (Å^2^)

**Table:**

	*U*^11^	*U*^22^	*U*^33^	*U*^12^	*U*^13^	*U*^23^
C1	0.0321 (10)	0.0470 (13)	0.0348 (10)	0.000	0.0025 (8)	0.000
C2	0.0351 (7)	0.0290 (8)	0.0338 (7)	−0.0026 (6)	0.0018 (5)	0.0010 (6)
C3	0.0459 (9)	0.0255 (7)	0.0369 (8)	−0.0028 (6)	0.0061 (6)	−0.0007 (6)
C4	0.0448 (12)	0.0348 (11)	0.0273 (9)	0.000	0.0094 (8)	0.000
C5	0.0565 (10)	0.0380 (9)	0.0412 (9)	0.0012 (7)	0.0069 (7)	0.0031 (7)
C6	0.0521 (10)	0.0540 (11)	0.0472 (10)	0.0090 (8)	0.0080 (8)	0.0093 (8)
C7	0.0445 (13)	0.0660 (16)	0.0329 (11)	0.000	0.0123 (9)	0.000
C8	0.0496 (16)	0.095 (2)	0.0456 (14)	0.000	0.0063 (11)	0.000
N1	0.0482 (11)	0.0259 (9)	0.0334 (9)	0.000	0.0073 (7)	0.000
O1	0.0641 (8)	0.0342 (6)	0.0340 (6)	−0.0022 (5)	0.0046 (5)	−0.0071 (4)
S1	0.0498 (3)	0.0270 (3)	0.0257 (3)	0.000	0.0050 (2)	0.000
S2	0.0525 (3)	0.0367 (3)	0.0409 (3)	−0.00501 (17)	0.01189 (18)	0.00431 (15)
S3	0.0545 (4)	0.0717 (5)	0.0429 (4)	0.000	0.0168 (3)	0.000

### Geometric parameters (Å, º)

**Table:**

C1—S3	1.635 (2)	C5—H5	0.9300
C1—S2	1.7423 (17)	C6—C7	1.391 (3)
C1—S2^i^	1.7423 (17)	C6—H6	0.9300
C2—C3	1.356 (2)	C7—C6^i^	1.391 (3)
C2—C2^i^	1.422 (3)	C7—C8	1.498 (4)
C2—S2	1.7359 (16)	C8—H8A	0.9600
C3—N1	1.391 (2)	C8—H8B	0.9600
C3—H3	0.9300	C8—H8C	0.9600
C4—C5^i^	1.385 (2)	N1—C3^i^	1.391 (2)
C4—C5	1.385 (2)	N1—S1	1.679 (2)
C4—S1	1.747 (3)	O1—S1	1.4221 (14)
C5—C6	1.376 (3)	S1—O1^i^	1.4221 (14)
			
S3—C1—S2	122.58 (7)	C6—C7—C8	120.86 (12)
S3—C1—S2^i^	122.58 (7)	C6^i^—C7—C8	120.86 (12)
S2—C1—S2^i^	114.85 (14)	C7—C8—H8A	109.5
C3—C2—C2^i^	108.56 (9)	C7—C8—H8B	109.5
C3—C2—S2	135.53 (13)	H8A—C8—H8B	109.5
C2^i^—C2—S2	115.87 (6)	C7—C8—H8C	109.5
C2—C3—N1	106.24 (15)	H8A—C8—H8C	109.5
C2—C3—H3	126.9	H8B—C8—H8C	109.5
N1—C3—H3	126.9	C3—N1—C3^i^	110.37 (18)
C5^i^—C4—C5	121.4 (2)	C3—N1—S1	123.26 (10)
C5^i^—C4—S1	119.27 (11)	C3^i^—N1—S1	123.26 (10)
C5—C4—S1	119.27 (11)	O1—S1—O1^i^	120.99 (11)
C6—C5—C4	118.71 (18)	O1—S1—N1	105.49 (7)
C6—C5—H5	120.6	O1^i^—S1—N1	105.49 (7)
C4—C5—H5	120.6	O1—S1—C4	110.02 (7)
C5—C6—C7	121.42 (19)	O1^i^—S1—C4	110.02 (7)
C5—C6—H6	119.3	N1—S1—C4	103.14 (10)
C7—C6—H6	119.3	C2—S2—C1	96.66 (9)
C6—C7—C6^i^	118.3 (2)		

Symmetry code: (i) *x*, −*y*, *z*.

### Hydrogen-bond geometry (Å, º)

**Table:**

*D*—H···*A*	*D*—H	H···*A*	*D*···*A*	*D*—H···*A*
C3—H3···O1^ii^	0.93	2.33	3.243 (3)	166

Symmetry code: (ii) −*x*+1/2, −*y*+1/2, −*z*+2.
